# Pre-pandemic Psychobiological Features Predict Impact of COVID-19 Confinement on Loneliness

**DOI:** 10.3389/fpsyg.2022.874232

**Published:** 2022-04-28

**Authors:** Shishir Baliyan, José Manuel Cimadevilla, Matias M. Pulopulos, Leyre Castillejo, Carmen Sandi, César Venero

**Affiliations:** ^1^COGNI-UNED, Department of Psychobiology, Faculty of Psychology, UNED, Madrid, Spain; ^2^Department of Psychology, University of Almería, Almería, Spain; ^3^Department of Experimental Clinical and Health Psychology, Ghent University, Ghent, Belgium; ^4^Laboratory of Behavioral Genetics, Brain Mind Institute, École Polytechnique Fédérale de Lausanne, Lausanne, Switzerland; ^5^Instituto Mixto de Investigación-Escuela Nacional de Sanidad, Madrid, Spain

**Keywords:** loneliness, social confinement, COVID-19, social loneliness, extraversion, cortisol, empathy, personality

## Abstract

The coronavirus disease 2019 (COVID-19) pandemic led to various government-imposed limitations on social interaction and strict home confinement. Such involuntary social-distancing policies can exacerbate feelings of loneliness and alter emotional well-being. Dysregulation of the hypothalamic-pituitary-adrenocortical (HPA) axis is a potential mechanism for loneliness’ deleterious health effects. In this study, we explored whether pre-pandemic diurnal cortisol output (AUC_*G*_), a measure of HPA axis function, may predict the propensity to changes in loneliness during long-term COVID-19 home confinement and if extraversion would moderate this relationship. This association has been explored by analysing the impact of COVID-19 pandemic and strict home confinement on social and emotional loneliness in 45 Spanish young adults. Diurnal cortisol levels were measured from five saliva samples obtained across a day just before the pandemic, and data about participants’ perceived loneliness, empathic state, extraversion, and prospective volunteering were obtained both before and during the confinement. Participants’ social and family loneliness increased during long-term strict home confinement, while prospective volunteering tendencies and extraversion decreased. Importantly, after adjusting for relevant confounders, moderation analyses revealed that in young adults with high pre-pandemic extraversion, a higher AUC_*G*_ predicted a larger increase in social loneliness during confinement, while in individuals with low extraversion, AUC_*G*_ was negatively related to change in loneliness. Our findings highlight the utility of pre-pandemic diurnal cortisol output in predicting the social impact of COVID-19 home confinement, presenting this hormone as a potential biomarker for *a priori* identification of at-risk groups during public health crises.

## Introduction

Loneliness is a complex unpleasant feeling rooted in a state of mind in which one’s interpersonal relationships are perceived as inadequate (Peplau and Perlman, 1982). This perception of inadequate interpersonal relationships is a result of a discrepancy between the quality and/or the quantity of desired and actual social connections. Loneliness has been proposed to be composed of social and emotional dimensions (Weiss, 1973). Social loneliness results from a sense of dissatisfaction with one’s general social life and interactions, while emotional loneliness consists of dissatisfactory intimate emotional ties, such as with a spouse, parent, or sibling. Loneliness can affect all age groups, but its prevalence is higher in young (18–30 years) and elderly (>80 years) adults (Hawkley et al., 2022). As a consequence of the COVID-19 pandemic, governments across the globe had and are re-enforcing population confinements. Subjects of the current study underwent a strict, 50-day lockdown with citizens obliged to remain inside their residence except for the purchase of essential commodities or if in need of medical assistance. Such extensive confinement accompanied by overhauling changes to daily social interactions demand more attention for two main reasons; firstly, because recent studies have reported sharp increments in feelings of loneliness following lockdowns (Groarke et al., 2020); and secondly, because it is well known that long-term loneliness increases the risk of detrimental health consequences, including higher blood pressure, anxiety, depression and all-cause mortality (Cacioppo and Cacioppo, 2014; Martín-María et al., 2020). Although the impact of the pandemic and confinement on loneliness has made things harder across different populations such as healthcare workers (Mansueto et al., 2021), evidence suggests that younger adults (18–24 years) merit specific attention for being the group most hard-hit (Groarke et al., 2020; Sampogna et al., 2021).

There is evidence indicating the existence of diverse neural, metabolic, endocrine and genetic factors that seem to mediate the association between loneliness and health outcomes. Specifically, loneliness was found to be associated with enhanced inflammation, high blood pressure, coronary heart disease and strokes, diminished immunity and higher rates of metabolic syndrome (Pressman et al., 2005; Hawkley et al., 2006; Holt-Lunstad and Smith, 2016; Leigh-Hunt et al., 2017; Donovan and Blazer, 2020). In addition, loneliness is considered a psychosocial stressor that has been associated with a dysfunction of the HPA axis (Steptoe et al., 2004; Doane and Adam, 2010). Alterations in the diurnal activity of the hypothalamic-pituitary-adrenocortical (HPA) axis, the neuroendocrine network integral to the biological stress system, have also been associated with poor health (Hawkley et al., 2012). In this regard, cortisol, the primary stress hormone and end product of the HPA axis, is an important predictor of future health problems among youth (Adam et al., 2017). Adrenal release of cortisol follows a circadian pattern with highest cortisol levels in the morning and a subsequent decline throughout the day, with total diurnal cortisol release estimated as area under the curve with respect to ground (AUC_*G*_) (Pruessner et al., 2003). In the last decade, a dysregulation of the HPA axis has been proposed as a potential mechanism through which loneliness-led heightened social threat perception may trigger pernicious health effects (Cacioppo et al., 2002; Miller and O’Callaghan, 2002; Cacioppo and Hawkley, 2009). The literature concerning the association between loneliness and diurnal cortisol pattern in young-adult samples have reported inconsistent results. Thus, in some studies, loneliness feelings were found related to higher AUC_*G*_ (Pressman et al., 2005; Lai et al., 2018). In contrast, other studies found either no relationship between loneliness and cortisol (Cacioppo et al., 2002), a flattening of the diurnal cortisol rhythm (Doane and Adam, 2010), or even a smaller cortisol awakening response (Jopling et al., 2021). Although these studies in young-adult samples show contrasting results, they suggest an important relation between cortisol and loneliness.

A growing body of evidence points toward loneliness as not only a cause of, and to correlate with stress, but also a possible consequence of biological factors, like individual differences in cortisol output (Cacioppo and Hawkley, 2009; Campagne, 2019). In a large-cohort longitudinal study, Stone et al. (2013) showed that living alone was not a risk factor for poor mental health, but the stress of the transition to living alone was crucial for symptoms of clinical loneliness. Critically, Cole et al. (2015) showed that the conserved transcriptional response to adversity (CTRA) (upregulated inflammation and downregulated anti-viral gene expression, hallmark of chronic stress) preceded loneliness. Similarly, reduction of stress via mindful meditation, known to alter cortisol output (Brand et al., 2012), has been shown to reduce loneliness as well (Creswell et al., 2012; Lindsay et al., 2019). Although some of the studies mentioned above only implicate a possible dysregulation of the HPA axis, and not cortisol *per se*, to be related to loneliness, we consider exploration of individual diurnal cortisol output, a measure of HPA axis function, to be a robust first step to explore that relation.

Individual personality differences are also known to play a crucial role in this interdependent relationship between loneliness and stress, given how the need for social connections or the impact/threat of their loss – as by current pandemic and the confinement periods – varies from person to person (Cacioppo and Patrick, 2008). Multiple cross-sectional studies in young adults have reported that loneliness relates inversely to extraversion (Stokes, 1985; Cheng and Furnham, 2002; Cacioppo et al., 2006; Atak, 2009; Buecker et al., 2020). Extraversion has a strong social implication as it involves the tendency to socially approach others and to enjoy their company. Individuals high in extraversion can make new friends more easily and be more satisfied with their social interactions compared to individuals low in extraversion (Wagner et al., 2014). According to Eysenck’s theory of personality (Eysenck, 1967), extraversion is associated with lower levels of cortical arousal which leads to a greater need for stimulation. This need is in turn met by behaviours that increase the type and degree of interpersonal and social relations. Following the results of their study and interpreting them in light of Eysenck’s theory, Saklofske and Yackulic (1989) discussed how *“for the extravert, loneliness may occur when limitations are placed on the opportunity to interact with others on a regular basis”* (p. 4). It has been reported that extraversion relates to more positive objective life events (Magnus et al., 1993) and aids attention to positive aspects of stressors (Hemenover and Dienstbier, 1996). Low extraversion has been associated with both, low and high basal cortisol outputs (Hauner et al., 2008; Laceulle et al., 2015; Ouanes et al., 2017; Limone et al., 2021). In a study using young-adult samples, researchers found extraversion to negatively predict cortisol responses to stress (Pérez et al., 2004; Xin et al., 2017), while in older adults, extraversion was also found to be negatively related to AUC_*G*_ (Ouanes et al., 2017), indicating a relationship between extraversion and the HPA system. A recent study by Erickson et al. (2021) presented the relationship between extraversion and total cortisol output to be curvilinear (and inverted U shape). In addition, Lu et al. (2014) observed that extraversion moderates the relationship between loneliness and peer attachment, a measure that has been associated with interindividual differences in HPA axis activity (e.g., Hicks and Diamond, 2011; Kidd et al., 2013; Pietromonaco and Powers, 2015).

**FIGURE 1:**
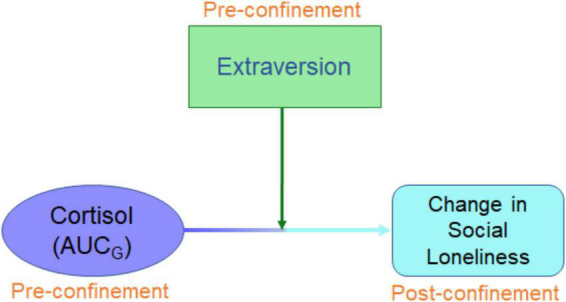
Conceptual diagram of working hypothesis. Influence of pre-pandemic cortisol on change in social loneliness will be moderated by extraversion.

Thus, the strict social confinements due to the COVID-19 pandemic present a salient opportunity for studying, outside laboratory settings, the existence of a relationship between total cortisol output and the propensity to experience changes in loneliness. Given the aforementioned evidences of the relationship between cortisol AUC_*G*_ and loneliness as well as AUC_*G*_ and extraversion, we decided to use this cortisol AUC_*G*_ index. Remarkably, this is the most commonly used cortisol index in research studies, and it captures both intensity (overall distance of cortisol samples from the ground) and sensitivity (difference between individual cortisol samples) reducing the difficulties in analysing datasets containing repeated measures of cortisol (Pruessner et al., 2003). Concerning extraversion and loneliness during the COVID-19 lockdown, the literature has yielded mixed effects. Some studies indicated that extraversion is a protective factor against loneliness (Tutzer et al., 2021) while others reported that extroverts are not so likely to engage with social distancing and confinement measures (Carvalho et al., 2020), probably because they seek social proximity. Accordingly, it would be reasonable to examine if the relationship between AUC_*G*_ and social loneliness differs when extraversion varies between individuals (Figure 1). However, since no previous research has investigated the relationship between cortisol, loneliness (social or family), and extraversion, we did not have a specific directional hypothesis of how extraversion would interplay with cortisol and loneliness. Finally, the impact of the COVID-19 pandemic and the following confinement on the self-perception of loneliness, extraversion, state empathy and prospective volunteering were studied across pre-pandemic and during-strict-confinement timepoints. The relation between loneliness and empathy was of interest given their negative correlation among younger adults and the possible impact a forced lockdown may have on it (Davis, 1983; Kalliopuska, 1986). The principal reason for testing prospective volunteering was to discriminate between expected stability in or even an increase in empathetic scores and a possible decrease in prosocial tendencies.

## Materials and Methods

### Procedure

**FIGURE 2:**
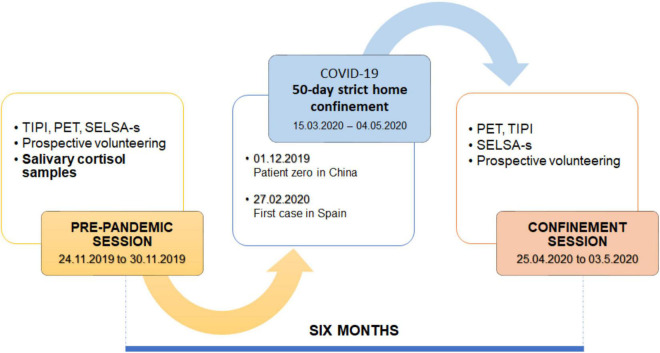
Study timeline. SELSA-S, Social and Emotional Loneliness Scale-Short; PET, Pictorial Empathy Test; TIPI, Ten-Item Personality Index.

This research was conducted across two time points, pre-pandemic (24.11.2019 to 30.11.2019) and during-confinement (24.4.2020 to 30.4.2020) (Figure 2). The first step at both time points was obtaining relevant ethics committee approval (UALBIO2020/020) and obtaining informed consent from participants.

All procedures complied with specifications outlined by the European Communities Council Directive 2001/20/EC and the Helsinki Declaration for biomedical research involving humans.

### Participants

First-year university students were invited to take part in a study investigating personality. Seventy-nine students participated and study sample characteristics are listed in Table 1 (78% females; mean age 20.68; SD 5.19). Following receival of the invitation to the second assessment, 45 of these students consented and their data were collected during home confinement. The students received course credit for participating in this study. No differences in the variables investigated in this study were observed between subjects who did not participate in the second assessment and the final sample included in the current investigation.

**TABLE 1:** Characteristics of the study sample for cohort that participated in both sessions of the study.

	*Mean (SD)*
Sex	80% female
Age	21.09 (6.42)
Ethnicity	93.6% Caucasian
AUC_*G*_	3.880 (1.492)

*SD, standard deviation; AUC_G_ (μg/dL), total diurnal cortisol release.*

### Cortisol

During pre-pandemic assessment, participants were given saliva sample collection vials (Salivette^®^, Sarstedt) along with detailed verbal and written instructions concerning sample collection. Subjects collected five samples, at 0, 30, and 45 min after awaking, 7 h following awakening and right before sleeping. Relevant health conditions, oral contraceptives, previous history of mental ailment or medication impacting either cortisol or mental state, and improper/ill-timed sample collection (>±5 min) were employed as exclusion criteria. Four subjects’ sample collection times deviated +5 min from the expected time point during the post-awakening measures, five participants did not provide all the saliva samples and two participants provided saliva samples with haemic contamination, so they were excluded from the analyses. Diurnal cortisol output was calculated as the area under the curve with respect to the ground (AUC_*G*_). Cortisol levels were analysed via a commercially available enzyme-linked immunosorbent assay (Salimetrics^®^) having a sensitivity of <0.007 μg/dL with inter- and intra-assay precision of 5.1 and 3.2%, respectively.

### Questionnaire Measures

#### Social and Emotional Loneliness Scale for Adults-Short

Social and Emotional Loneliness Scale for Adults-Short (Yaben, 2008) measures three distinct facets of loneliness via its two subscales of social loneliness and emotional loneliness. The emotional loneliness subscale is further broken down into romantic loneliness and family loneliness. The items consist of descriptions of feelings and subjects are instructed to mark how accurate those descriptions are for themselves using a 7-point Likert-type scale. Scale reliability; McDonald’s ω pre-pandemic = 0.82 and during-confinement = 0.84.

#### Ten Item Personality Inventory

Ten Item Personality Inventory (Romero et al., 2012) was used to quantify extraversion during both sessions of the study. The TIPI consists of items representing characteristics of personality and subjects are asked to mark how well each item describes them on a 7-point Likert-type scale. Scale reliability; McDonald’s ω pre-pandemic = 0.81 and during confinement = 0.68.

#### Pictorial Empathy Test

The Spanish version of the PET (Baliyan et al., 2022) was used to quantify situational emotional empathy during both time points of assessment. PET consists of seven photographs of people in suffering, each image followed by inquiry of the immediate empathic reaction of the subject to said stimuli using a 5-point Likert-type scale. Scale reliability; McDonald’s ω pre-pandemic = 0.77 and during confinement = 0.79. PET and volunteering were explored in-person during the pre-pandemic session and online at the during-pandemic session while loneliness and extraversion were assessed online during both, pre-pandemic and during-confinement sessions.

#### Prospective Volunteering

After completing PET, subjects were asked to answer, assuming having available 30 days of vacations, how many days would they dedicate to volunteering with a non-government organisation working to aid people in suffering. During confinement subjects again underwent the PET and answered the same question about prospective volunteering. This procedure was based on previous research concerning subject responses to distressing images and related time contribution to volunteering (Burt and Strongman, 2005).

### Data Management and Statistical Analyses

For cortisol levels, we calculated total diurnal cortisol output via the area under the curve with respect to the ground, plotting each individual subject’s cortisol samples collected at their respective awakening, +30 min, +45 min, 7 h following awakening and at their respective bedtime (Pruessner et al., 2003). The changes in values of variables of interest were estimated by subtracting the confinement session scores from the pre-pandemic scores thus allowing us to calculate the extent of the changes which were then used as dynamic variables. Thus, Wilcoxon’s signed ranks test was utilised to investigate changes in loneliness, state empathy, volunteering tendencies and extraversion from pre-pandemic to values during confinement. Correlations were used to explore the association among the quality of relationships in the house, number of cohabitants, and the aforementioned variables, the significance level was set at *p* ≤ 0.05, two-tailed, for all analyses. To run our proposed moderation models, we utilised regression-based path analysis via the PROCESS plugin (version 3.5) for SPSS. PROCESS is a macro to estimate and probe interactions (Hayes, 2017). We estimated model 1 for moderation (working hypothesis) in PROCESS using 5000 bootstrap samples and 95% bias-corrected bootstrap confidence intervals. In the moderation model, we tested whether AUC_*G*_ related to changes in social loneliness as measured using SELSA-S, while being moderated by prior extraversion scores. Effect sizes are provided as standardised coefficients (β) but unstandardised coefficients are also provided for the moderation analyses to provide readers with the opportunity to interpret raw scores. No uni- or multivariate outliers were found among the variable values used in the analyses. All statistical analyses were carried out through the statistical package for social sciences (SPSS) version 25.0 (IBM, Armonk, NY, United States).

## Results

### Impact of the Pandemic and Home Confinement on Loneliness, State Empathy, and Extraversion

**FIGURE 3:**
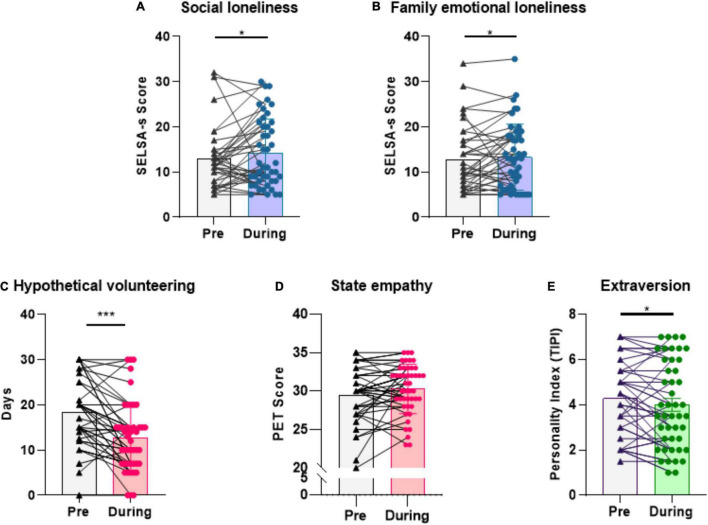
Pre-confinement and during-confinement scores. Bar graphs showing **(A)** an increase in Social loneliness **(B)** an increase in Family emotional loneliness **(C)** a decrease in number of days dedicated to a hypothetical volunteering choice **(D)** no change in scores on state empathy and **(E)** a decrease in extraversion scores as measured by the TIPI. SELSA-S, Social and Emotional Loneliness Scale-Short; PET, Pictorial Empathy Test; TIPI, Ten-Item Personality Index. *p < 0.05; ***p < 0.001.

Pre-pandemic values were compared to during-lockdown values using Wilcoxon’s signed ranks tests for measures of social and family loneliness, perspective volunteering tendencies, the pictorial empathy test and extraversion (Figure 3). We observed a significant increase in family and social loneliness (*z* = −2.031, *p* = 0.04 and *z* = −2.5089, *p* = 0.04, respectively). Specifically, family loneliness scores increased from 12.6 to 14.1 while social loneliness scores increased from 12.8 to 14.9. Regarding measures of empathy, we observed a significant decrease in the total number of days offered volunteering during confinement, while no significant change was observed among PET scores (*z* = −4.294, *p* < 0.01; *z* = −1.846, *p* = 0.07). Trait extraversion decreased post-confinement (*z* = −2.001, *p* = 0.04).

### Unadjusted Correlations

Spearman correlation coefficients between study variables are displayed in Table 2. Among the most important correlations, we observed that the quality of the relations with cohabitants negatively correlated with change in family loneliness (*r* = −0.32, *p* = 0.017). Interestingly, change in social loneliness correlated with change in volunteering duration, such that increase in volunteering corresponded with a higher increase in social loneliness (*r* = 0.43, *p* < 0.001).

**TABLE 2:** Unadjusted correlation matrix.

	*Residence change*	*QRC*	*During volunteering*	*Pre extraversion*	*During extraversion*	*Pre F.L*	*Pre S.L*	*During F.L*	*During S.L*	*Pre PET*	*Post PET*	*ΔVolunteering*	*ΔFamily loneliness*	*ΔSocial loneliness*	*ΔPET*
*QRC*	0.069														
*During volunteering*	0.295*	–0.204													
*Pre-extraversion*	0.353*	0.061	0.195												
*During extraversion*	0.311*	0.104	0.115	0.813**											
*Pre-family loneliness*	–0.268	–0.270	–0.296	–0.177	–0.220										
*Pre-social loneliness*	–0.073	0.205	−0.326*	–0.214	–0.191	0.582**									
*During family loneliness*	–0.130	−0.510**	0.014	–0.132	–0.091	0.627**	0.160								
*During social loneliness*	−0.318*	–0.162	–0.013	−0.518**	−0.530**	0.451**	0.502**	0.537**							
*Pre-PET*	–0.123	–0.011	0.252	0.055	–0.071	–0.131	−0.303*	–0.138	–0.003						
*During-PET*	–0.044	0.234	0.169	0.001	–0.116	–0.247	–0.255	–0.243	0.086	0.592**					
*ΔVolunteering*	–0.004	–0.107	0.462**	–0.079	–0.132	0.065	–0.006	0.179	0.284	0.185	0.125				
*ΔFamily loneliness*	0.152	−0.317*	0.418**	0.101	0.065	−0.324*	–0.215	0.478**	0.066	–0.164	0.051	0.257			
*ΔSocial loneliness*	–0.244	–0.209	0.268	−0.368*	−0.513**	0.220	–0.173	0.327*	0.672**	0.343*	0.425**	0.427**	0.128		
*ΔPET*	0.062	0.166	–0.024	–0.114	–0.067	–0.195	–0.088	–0.132	0.003	−0.494**	0.321*	0.112	0.217	0.153	
*AUC*_*G*_	–0.065	0.203	0.181	–0.106	0.047	–0.288	–0.225	–0.171	–0.125	–0.131	0.165	0.075	0.273	–0.001	0.321*

*AUC_G_, cortisol index for area under curve from ground; QRC, quality of relationships with cohabitants; F.L, family loneliness; S.L, social loneliness; Δ, change in score calculated as pre-pandemic values subtracted by during conferment scores for the respective variables; PET, Pictorial Empathy Test.*

Spearman coefficients reported; *p < 0.05; **p < 0.01.

*Significant values are presented in bold.*

### Moderation Analyses

Using path-analysis models, we investigated our working hypothesis (Figure 1); whether pre-pandemic diurnal cortisol output and post-confinement change in social loneliness had a relationship, which was moderated by extraversion. Sex, residence change during confinement, and the number and the quality of relations with cohabitants were covariables controlled for. The pattern of results did not differ on exclusion of all covariates, underscoring the robust association between the variables of interest.

#### AUC_*G*_ With Pre-extraversion as Moderator

**FIGURE 4:**
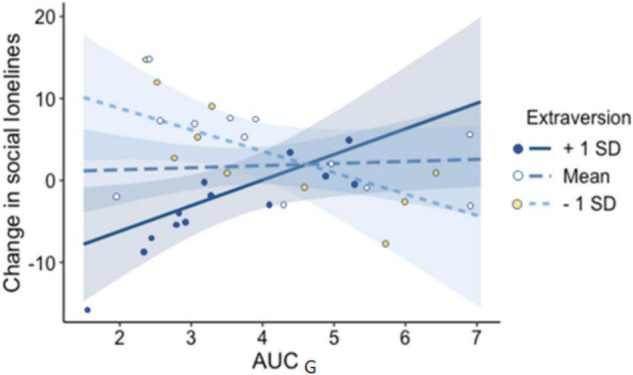
Simple slopes plots of conditional effects representing the association between pre-Extraversion and pre-lockdown cortisol AUC_*G*_. AUC_*G*_, cortisol index for area under curve from ground; SD, standard deviation.

The overall model was significant *F*(7,34) = 2.90, *p* = 0.02, showing that 44% of the variance in change in social loneliness was predicted by AUC_*G*_, pre-extraversion and their interaction. AUC_*G*_ and pre-extraversion’s interaction significantly predicted change in social loneliness (AUC_G_*pre-extraversion: β = 0.52, *p* = 0.01). Simple slopes (at mean and ±1 SD pre-extraversion score) are presented in Figure 4. Johnson-Neyman significance regions analysis revealed that when pre-extraversion is more than 5.62 (β = 0.83), AUC_*G*_ and change in social loneliness are significantly positively related, *b* = 2.40; β = 0.47, *p* = 0.05. However, at and below low extraversion scores (<2.5) AUC_*G*_ and change in social loneliness are negatively and significantly related *b* = −4.0; β = −0.75, *p* = 0.05. Moderation models with change in family loneliness were not statistically significant.

## Discussion

This study examines the prospective association between cortisol, social and family loneliness, and extraversion. It documents the impact of COVID-19 pandemic confinement in association with biological markers of stress and attends to possible psychobiological features relevant to the identification of vulnerable groups. Firstly, via a longitudinal study, we examined the impact of the pandemic confinement on loneliness in young adults. Our results showed that, during long-term home confinement, most subjects reported increased feelings of social and family loneliness. The pandemic and confinement’s effects were also reflected in participants reporting themselves to have lower extraversion trait-like characteristics and a substantial decrease in prosocial tendencies, as evidenced by the diminished prospective volunteering. Importantly, individual differences in pre-pandemic total output of cortisol (AUC_*G*_) were able to predict the impact of strict social confinement on social loneliness, an association moderated by pre-pandemic extraversion scores, affecting the intensity and direction of the relation.

As the feelings of loneliness may be influenced by the students having had to change their primary residence as a result of the forced long-duration confinement, or by the number and/or quality of relations with the cohabitants they shared their residence with, we considered these variables as potential confounders. Also, given sex differences concerning loneliness scores across young adults (Borys and Perlman, 1985; Cramer and Neyedley, 1998) and specifically during COVID-19 (McQuaid et al., 2021), we added sex as another covariate in all relevant analyses. Interestingly, the results of the current study complement the results obtained by another longitudinal study concerning the relationship between loneliness and cortisol, also carried out during the COVID-19 pandemic. Across 52 early-adolescent youth, Jopling et al. (2021) found that the pandemic-led increase in loneliness was associated with higher awakening cortisol levels. The apparent impact of increased loneliness-stress on cortisol production observed in that study, when paired with our results of diurnal cortisol output together with extraversion predicting change in loneliness, points toward a possible bi-directional relationship between loneliness and the HPA-axis in adolescent and young adult individuals. However, further studies are required to understand better the impact of loneliness on HPA axis.

In a previous study, higher extraversion was related to lower CAR (van Santen et al., 2011), while in older adults, lower extraversion was related to elevated diurnal cortisol output (AUC_*G*_) (Ouanes et al., 2017). In the present study, we observed that most subjects with higher pre-pandemic extraversion levels showed a reduction in loneliness during the pandemic, an effect that may be related to strong social support when encountering stressors (Swickert et al., 2002). Findings of some previous studies had indicated that, compared to introverts, extraverted individuals experienced higher stress levels (Liu et al., 2021; Zacher and Rudolph, 2021) and larger declines in social connectedness (Folk et al., 2020). However, extraversion has also been shown to be related to lower perceived stress and better emotional regulation (Barańczuk, 2019) and to be a protective factor against anxiety during the COVID-19 pandemic (Morales-Vives et al., 2020; Nikčević et al., 2021). This association, where extraversion relates to perceived stress, both positively and negatively, fits with the results of the current study and the moderating role of extraversion. The complex nature of loneliness not only draws attention to the fact that individuals vary in their quantity and quality of relationship needs, but also that distinct types of personality fulfil these needs dissimilarly. Thus, overall, the presented moderation model highlights how diurnal cortisol output and personality type (highly social or personalities more tolerant of isolation) shape individual differences in sensitivity to restricted social contact. Additionally, the finding that extraversion pivots the increase or decrease of perceived change in social loneliness during home-confinement at different pre-pandemic diurnal cortisol levels may help to understand the -sometimes contradictory- results reported across studies exploring the relationship between cortisol and loneliness.

According to the evolutionary theory of loneliness, perceived lack in quantity and/or quality of intimacy or companionship motivates one to make new or strengthen existing social connections (Cacioppo et al., 2006). While we did not see any changes in state/situational emotional empathy, as measured by the PETs, nor in trait emotional empathy, as reported earlier in a previous study from the same research project (Baliyan et al., 2021), we observed a strong decrease in prospective volunteering intentions. Given that the viral pandemic and imposition of lockdown specifically prevented social contact, working with strangers via a charitable organisation for helping strangers can be less appealing owing to the heightened risk of viral contagion. For most people, being quarantined is a stressful experience that increases anxiety and depressive symptoms (for rev. see Brooks et al., 2020). Therefore, it may be speculated that the negative psychological impact of quarantine and of a highly contagious virus (Brooks et al., 2020) may affect the appeal of volunteering, which itself is associated with possible psychological dangers like exhaustion, nervousness, and depression (Capner and Caltabiano, 1993; Mitchell et al., 2004). Additionally, while pre-pandemic there was an expected significant positive correlation between prospective volunteering and PET scores, this correlation was lost during confinement, likely due to the reasons mentioned above and not due to changes in empathy-like characteristics. Therefore, given the circumstances, we consider our results about emotional empathy and prosocial behaviour to not be at odds with one another. Interestingly, in the correlation results, we also noticed that those who suffered greater worsening in social loneliness were willing to spend more time volunteering. Thus, subjects who had experienced greater social loneliness also had stronger motivation to increase their social interactions and, perhaps, attend to the heightened feeling of inadequate social connections and reduce their social pain.

Change in social loneliness has a strong inverse relationship with individuals’ extraversion score during confinement. Previously, Cacioppo et al. (2006) not only showed loneliness to be related to extraversion, but also found that high loneliness is associated with greater shyness and lesser sociability among young adults. Cheng and Furnham (2002) showed how extraversion had direct and indirect effects on loneliness, while Mund and Neyer (2016) carried out a 15-year longitudinal study to show loneliness predicted future development, even magnitude, of extraversion traits. Here, we also observed that strict social confinement during COVID-19 pandemic reduced extraversion scores. Specifically, social loneliness has been found to be more strongly associated with low extraversion than emotional loneliness (Buecker et al., 2020). Extraversion is generally associated with being cheerful, optimistic, preferring social encounters, and experiencing more daily positive emotions (McCrae and Costa, 2003). While personality dimensions are generally stable and withstand major life events (Specht et al., 2011), there is also research into the temporary effects of depression and anxiety disorders on personality (Karsten et al., 2012), as well as the more lasting impact of interventions (Roberts et al., 2017). In fact, Sutin et al. (2020) also observed changes in personality (lower neuroticism) following the COVID-19 pandemic. Our view is that the decrease in extraversion observed in our study reflects situation-caused personality states changing one’s self-perception (Fleeson, 2001, 2004, 2007). Nonetheless, while trait measures could be expected to revert to their previous values when the immediate acute pandemic situation has ended, our data strongly support the need for future longitudinal studies on the topic to include personality assessments. Finally, our pre-pandemic observations did replicate the negative relation between loneliness and empathy as reported previously (Beadle et al., 2012). However, subsequent during-confinement exploration of the same relation revealed that the association had been lost. The asynchronisation of this relationship may be due to the abrupt and “forced” nature of the increase in loneliness which did not coincide with a corresponding decrease in empathetic tendencies.

Overall, we can appreciate the fact that some limitations should be considered when interpreting the results of this study. Given the circumstances, the sample size of the study was constrained, and it was not possible to collect saliva samples during the confinement. Moreover, it was not feasible to have a control group not submitted to the lockdown. The majority of the participants were female, and the recruitment procedure leaves the results open to sample selection bias. Additionally, the study did not account for exposure to early-life trauma, which is known to potentially cause HPA-axis dysregulation affecting cortisol (Danese and McEwen, 2012; Faravelli et al., 2017). Nevertheless, we deem our results highly informative, given how few studies involving natural stressors of this kind are available and the inherent challenges to obtaining relevant data.

Our findings support the notion that the relationship between cortisol, extraversion, and loneliness might be more complex than expected and that diurnal cortisol might not always equate to health risk in certain populations. Future studies should focus on how total cortisol output can be used as a comprehensive means to identify individuals at risk for feelings of loneliness and explore proofs of causality. Although the current results underscore the impact of stress preceding the onset of changes in social loneliness, it is conceivable that premorbid and preliminary propensity to loneliness (cognition, physiology, and behaviour) may also unfavourably influence both extraversion and individuals’ perception of loneliness. To the best of our knowledge, this is the first study indicating that extraversion and cortisol output interact to predict changes in loneliness following stress. More research is needed to corroborate this finding using larger samples and other indicators of HPA-activity and personality. If the findings of the present study can be confirmed, cortisol output might be useful for psychiatrists, general practitioners, occupational health services, and primary care physicians as a non-invasive and painless biomarker to identify and assist young people at risk for developing stress-related loneliness.

## Conclusion

Loneliness as a stressor among the population must be monitored closely given that although transient loneliness promotes the desire to socialise, the failure to socialise risks entering a self-reinforcing loneliness feedback loop (Cacioppo and Hawkley, 2009), which can then be linked to HPA axis dysregulations. Given the continuously emerging waves of COVID-19 pandemic over the past couple of years, and the concomitant re-confinement measures frequently applied in many countries, social connections are under constant strain and, at the same time, they may be needed more than ever. Results from our study point toward the need of longitudinal studies exploring the transition of the state-of-mind into loneliness and explore possible biomarkers as prodromic or causal links underlying loneliness.

## Data Availability Statement

The raw data supporting the conclusions of this article will be made available by the authors, without undue reservation.

## Ethics Statement

The studies involving human participants were reviewed and approved by the Ethical Committee of Universidad de Almería (UALBIO2020/020). The patients/participants provided their written informed consent to participate in this study.

## Author Contributions

SB: conceptualisation, methodology, formal analysis, investigation, writing – original draft, writing – review and editing, data curation, and visualisation. JC: project administration, supervision, resources, and writing – review and editing. MP: visualisation, writing – review and editing, and investigation. LC: methodology, data curation, visualisation, and formal analysis. CS: supervision, resources, conceptualisation, and writing – review and editing. CV: supervision, conceptualisation, project administration, resources, and writing – review and editing. All authors contributed to the article and approved the submitted version.

## Conflict of Interest

The authors declare that the research was conducted in the absence of any commercial or financial relationships that could be construed as a potential conflict of interest.

## Publisher’s Note

All claims expressed in this article are solely those of the authors and do not necessarily represent those of their affiliated organizations, or those of the publisher, the editors and the reviewers. Any product that may be evaluated in this article, or claim that may be made by its manufacturer, is not guaranteed or endorsed by the publisher.
